# Public network attention to hiking in China and its influencing factors

**DOI:** 10.1371/journal.pone.0306726

**Published:** 2024-07-11

**Authors:** Qing Zhang, Huazhen Sun, Qiuyan Lin, Kaimiao Lin, Kim Mee Chong

**Affiliations:** 1 School of Tourism, Wuyi University, Nanping, China; 2 Graduate School of Business, SEGi University, Kuala Lumpur, Malaysia; 3 School of Accounting and Finance, Faculty of Business and Law, Taylor’s University, Subang Jaya, Malaysia; Covenant University, NIGERIA

## Abstract

In the process of hikers’ choosing a destination, searching for information is one of the important elements, playing a decisive role in decision-making. Based on the Baidu Index for “hiking,” this paper analyzes the spatial and temporal characteristics of and factors that influenced network attention to hiking in China from 2016 to 2021. The study found that (1) Network attention to hiking in China was generally relatively stable across the period studied, with highly uneven distribution between different months. The search volume was higher on weekends, and mobile searches increased year by year, far exceeding computer searches. (2) Different regions in China experienced different levels of network attention, with the highest levels in the east, followed by the center, and the lowest in the west. Except for East China, network attention to hiking was highly unevenly distributed within each region. (3) The COVID-19 pandemic increased the geographical concentration index and coefficient of variation but reduced the primacy index. A region’s level of economic development, degree of network development, population size, and population age structure are proposed as factors that affect network attention to hiking.

## 1. Introduction

The source market plays a crucial role in determining the viability and sustainable growth potential of tourism destinations, as well as in fostering competition among such destinations [1]. Research on the source markets of tourist destinations is widely conducted in the fields of tourism geography, tourism management, and marketing. Through this work, it has been extensively demonstrated that tourism destination branding, which is based on tourists’ perceived image of a location and that location’s potential appeal, is an effective marketing strategy adopted by many destinations [2–8]. According to the 51st report released by the China Internet Information Center, the number of Internet users in China has reached 1.067 billion as of December 2022, and the country’s Internet penetration rate is as high as 75.6%. Before embarking on their travels, tourists will gather their desired travel information, and the Internet serves as one of the simplest and most expeditious means to do so [7, 9, 10]. Broadly speaking, information search influences the decision-making process for purchases [11, 12]: The amount of information obtained and the sources from which people obtain information are thus strongly correlated with their travel intentions [13, 14]. Consumers expend money, time, and other resources on information consumption that may ultimately lead to a purchase [15–17]. Travelers gather information through their searches, and the records of this search process collectively constitute big data, thereby providing convenience to travelers seeking such information. The search volume of a keyword on a given search engine platform directly indicates the level of network attention [18]. Chinese residents who use the Internet mostly use the search engines offered by Baidu, Bing, and Google; around 72.4% of the market is owned by Baidu, making it the market leader [19]. Hence, the Baidu Index is a crucial big data measure for examining online focus in China. Big data has immeasurable value in the Internet age, and its impact is undeniable. In this paper, we aim to establish the meaning of this data for the topic of hiking.

In July 2021, the State Council of China explicitly called for “the development of a general plan for the construction of a national trail system,” and the State Forestry and Grassland Administration of China announced the establishment of three groups of 12 national forest trails. Hiking is one of the most popular and interesting outdoor hobbies [20–22] and one of the most fundamental activities undertaken in national parks and other outdoor recreation areas [23]. There are more than 100 million outdoor sports enthusiasts in China, and more than 20 million people have experienced hiking through the country’s forests (State Forestry and Grassland Administration Government Website, 2021). “Hiking network attention” refers to the index of web users’ attention to hiking, because hiking requires interested individuals to locate more relevant information than most other tourist activities. The analysis of hiking network attention can illustrate its temporal and spatial patterns and characteristics, which can provide insight and effective suggestions to attract hikers to tourist destinations.

Network attention indicators, such as Google Trends and the Baidu Index, employ data from online searches to gauge Internet users’ behavioral preferences and public opinion. Research on network attention was first conducted by Western scholars [24, 25], who focused mostly on investigating subjects such as search behavior and consumer decision-making processes using Google Trends [26–31]. Research has since revealed that Internet search data reflect the level of user interest in a particular phenomenon or object and that there is, to some extent, a correlation between this search data and users’ real-life social behavior [32]. With the support of network attention data, therefore, the geographical patterns of tourism flows can be clarified, as can the distribution of tourist sites, and innovative marketing techniques for scenic areas can be developed [33–35]. Research applications of network attention data have included work on tourism [27, 36–46], marketing strategies [3, 4, 47–51], and public events [52–58]. Chinese scholars employ the Baidu Index and data from other online search engines to predict the number of tourists visiting scenic sites, analyze problems with tourism security networks, assess passenger flow, and study the spatial distribution of concerns related to scenic sites [1, 59]. Fewer studies, however, have used network attention data to research hiking in China. There is, moreover, a general scarcity of studies focusing on Chinese hikers as subjects of research, as the majority of research conducted in this area has been centered on Western cultures [60].

Based on these gaps in published research, this study used several metrics to analyze spatial and temporal differences in network attention to hiking in 31 provincial-level administrative regions in China from 2016 to 2021. The main questions addressed were the following: (1) What are the spatial and temporal characteristics of network attention to hiking in China? (2) What is the difference in network attention to hiking among provinces in China? (3) What are the factors affecting public network attention to hiking in China? This paper presents the analysis, seeking to answer these questions, before finally proposing management approaches based on the findings for both companies and local governments.

## 2. Data sources and methodology

### 2.1 Data sources

The “Baidu Index,” often used for network attention analysis, is a tool based on Internet search analysis by the search engine Baidu that calculates the level of user attention and media attention for a given keyword. It can provide daily user search values, which can reflect trends in user attention to the provided keyword, and can present a demographic profile on demand of those users who searched for a keyword.

In this study, the keyword “hiking” was selected for entry into the Baidu Index, and given that the National Forestry Bureau of China decided in 2015 to begin construction of a national forest trail system, the period for study was set to that running from January 1, 2016, to January 1, 2022. The daily volume of web searches for “hiking” on personal computer and mobile terminals was obtained, as were monthly and yearly counts for each of the 31 provincial-level administrative regions (excluding Hong Kong, Macao, and Taiwan). These data were subsequently used to analyze the spatial and temporal characteristics of network attention to hiking in China.

The keyword selected is that which should best reflect and characterize users’ search behaviors and have a strong representativeness, a significant search volume, and a broad range of associated content. Unlike other, small-scale studies (such as provincial- and municipal-scale studies), where each hiker can be chosen as a keyword to search for, this study was conducted on a nationwide scale with tens of thousands of potential hikers involved. The term “hiking” has steadily evolved into a widely accepted concept in China, with both relevance and complexity of content behind it. As a result, while this study recognizes that using “hiking” as the sole keyword may have limitations, it is currently the most appropriate choice.

### 2.2 Methodology

Based on the data for network attention in 31 provincial-level administrative regions (excluding Hong Kong, Macao, and Taiwan) in China, this study draws on the method of regional economic difference analysis to analyze the spatial and temporal differences in network attention to hiking of network users across those 31 regions from 2016 to 2021 using the following six indicators:

1. Coefficient of variation (CV): This represents the ratio of the standard deviation to the mean. It is used to analyze and compare the degree of variation between sample indicators of economic scale in different regions [6]. The larger the CV, the more pronounced the difference between the network attention to hiking in each province and region in China.
$$CV=\sqrt{\frac{\sum_{i=1}^{n}{\left(x_{i}-\bar{x}\right)}^{2}}{n}}/\bar{x}$$
2. Herfindahl index (H): This is used to analyze the degree of agglomeration of regional economies. The greater the regional economic agglomeration, the closer the value of H to 1 [61]. Thus, the closer H is to 1, the higher the degree of regional agglomeration of network attention to hiking.
$$H=\overset{n}{\underset{i=1}{\sum }}p_{i}^{2}$$
3. Primacy index (P): This is used to compare the distribution of regional economies, reflecting the degree of economic concentration in a region, and is the ratio of the largest economic scale to the second largest economic scale [62]. The greater the value of P, the more concentrated and unbalanced the network attention to hiking within a region.
$$P=p_{1}/p_{2}$$
4. Geographic concentration index (G): The geographical concentration index is an important indicator reflecting the degree of concentration of public attention at the national scale [63]. It is used to analyze and compare the distribution of hiking network attention between different regions. The closer G is to 100, the more network attention to hiking is concentrated in a certain region.
$$G=100\times \sqrt{\overset{n}{\underset{i=1}{\sum }}{\left(p_{j}/p\right)}^{2}}$$
5. Seasonal concentration index (S): This is used to analyze and compare the temporal concentration of hiking network attention by month [64]. The closer S is to 0, the smaller the temporal variation of network attention to hiking, that is, the more even the monthly distribution is.
$$S=\sqrt{\overset{12}{\underset{i=1}{\sum }}{\left(x_{i}-8.33\right)}^{2}/12}$$
6. Evolution of the spatial pattern: ArcGIS10.2 software was used to produce a table of the evolution of the spatial pattern of network attention to hiking, allowing the analysis of spatial differences based on the calculated results.

## 3. Analysis of the spatial and temporal characteristics of network attention to hiking

### 3.1 Temporal differences

Across the period 2016–2021, as shown in Table 1, network attention to hiking remained relatively stable, except for 2020 and 2021, when, presumably due to the effects of COVID-19, it declined. Intra-annual trends varied. In 2016, network attention was at its highest in March, then gradually decreased from April to August before increasing again in September and October. In 2017, the index was relatively high from March to May, peaking in May, and remained at a stable, slightly lower level for the remainder of the year. In 2018, the level was relatively high from March to May, reaching its highest in April, and then, as in 2017, remained at a stable level. In 2019, network attention was relatively high from March to May, peaking in May, and then slowly decreased from June to September before rebounding in October. Due probably to COVID-19, 2020 saw lower numbers throughout the year, with an overall drop of around 20% compared to 2019; again, levels were relatively high for the year in April and May, although this year, they reached a maximum in September. Some improvement was recorded in 2021, which saw relatively high levels from March to May and a maximum in April, but overall levels were still lower than those in 2016–2019.

**Table 1:** Hiking network attention index by month in China, 2016–2021.

Month/parameters	2016	2017	2018	2019	2020	2021
1	18831	14752	20694	20128	12288	16637
2	16882	17842	14640	19239	11396	14892
3	30940	28480	27784	30664	17474	21401
4	29552	27806	28353	28682	19215	23327
5	28855	29633	27812	30973	19014	22102
6	22913	24763	23246	24731	17861	19010
7	19736	21032	20481	20225	18080	18092
8	19172	21066	20388	19488	19748	18794
9	20348	22591	18576	18775	17420	17048
10	20572	21065	23675	20128	17260	17080
11	19052	20786	21288	17056	17182	16891
12	18261	19242	21620	18258	18014	18606
Aggregate	265114	269058	268557	268347	204952	223880
S	1.760734	1.596445	1.455621	1.797031	1.20741	1.06424
H	0.087054	0.086392	0.085876	0.087209	0.085083	0.084692

An analysis of network attention to hiking by month across the whole period found that on average, February saw the lowest levels, with those levels hiking rapidly in March, peaking in April, and then gradually declining to their lowest point in November. When we considered the ratio of the average network attention for each month of the year across the six-year period to the average across all months in that period, we found that for the months between March and June, this ratio was greater than 1, with a maximum value of 1.27 in May and values of 1.25 in March, 1.26 in April, and 1.06 in June, making these four months the peak season for (network attention to) hiking. The ratios of 0.83 for January and 0.76 for February indicate these months as the low season. The S-values shown in Table 1, ranging from 1.0642 (2021) to 1.797 (2019) indicate an imbalance in the monthly distribution of network attention to hiking in China, with seasonal differences being apparent, although in 2020 and 2021, this index decreased considerably. Calculated using the traditional four seasons recognized in China, the aggregate network attention index for hiking during 2016–2021 was 354964 for the spring, 447848 for the summer, 351060 for the autumn, and 306036 for the winter, demonstrating a clear peak in the summer. Simultaneously, the H-value of network attention to hiking in China from 2016 to 2021 did not exceed 0.9, showing a highly uneven distribution across different months.

Further statistical analysis of network attention data from 2016 to 2021 was conducted with the daily counts classified by day of the week as a way to identify the weekly distribution characteristics of network attention levels. The results showed that the search volume was relatively stable from Monday to Friday and gradually increased moderately on the two weekend days, which may be attributable to many hikers’ conducting hiking activities on weekends and needing to search for information during these activities. In terms of how users searched for information, the use of mobile terminals in searches for “hiking” rose year by year, from 54% of such searches in 2016 to 77% in 2021.

### 3.2 Spatial differences

#### 3.2.1 Inter-provincial differences

We analyzed the differences in network attention to hiking between different regions of China from 2016 to 2021 by dividing our data according to the 31 provincial-level administrative regions directly under the central government, based on the IP addresses of users captured by the Baidu Index. The results of the analysis showed that there were significant spatial variations between different regions. As Table 2 shows, along with overall fluctuations across 2016–2021, there was a significant imbalance in the level of network attention to hiking among regions, with these levels being generally higher in the eastern region, while in the west, only Sichuan had a higher level, and all other regions saw levels lower than those of the eastern and central regions. The top 10 regions for network attention to hiking in 2016–2021 were Guangdong, Shanghai, Beijing, Jiangsu, Zhejiang, Sichuan, Shandong, Hubei, Fujian, and Henan. Compared by year, the rankings did not change substantially from 2016 to 2019, with Beijing and Jiangsu subsequently surpassing Shanghai and Fujian surpassing Hubei in 2020 and Jiangsu continuing its upward trend to rank second by 2021, while Zhejiang surpassed Beijing and Shanghai to rank third in the same year. By calculating the levels seen in the top 10 regions as a percentage of the national levels, we found that no major changes occurred over the six-year period, with this percentage growing incrementally from 46.12% to 46.77%.

**Table 2:** Spatial distribution of hiking network attention index, 2016–2021.

Region	2016	2017	2018	2019	2020	2021	Total	Sort by
Guangdong	78494	86127	89227	89577	71216	73383	488024	1
Shanghai	55640	58750	60841	59468	50490	51461	336650	2
Beijing	58281	57409	59128	57602	51702	52043	336165	3
Jiangsu	53969	56138	57746	57323	51403	54431	331010	4
Zhejiang	52139	54879	56378	56575	50977	53210	324158	5
Sichuan	51309	51015	52180	51886	47862	46595	300847	6
Shandong	47573	50058	52947	53340	47835	48786	300539	7
Hubei	43772	44763	46250	45363	39402	40879	260429	8
Fujian	42433	44243	44793	45118	41087	40257	257931	9
Henan	42182	44638	44549	45623	39431	39993	256416	10
Hebei	41374	41826	45367	45659	41039	40167	255432	11
Hunan	39436	41878	44242	43668	38786	39232	247242	12
Anhui	38856	40971	42199	43783	39539	38469	243817	13
Liaoning	40308	42455	43408	41421	34753	35118	237463	14
Chongqing	33795	37622	38818	38667	36000	33601	218503	15
Shaanxi	34408	36623	41282	39799	32760	33348	218220	16
Yunnan	35412	37124	39066	38172	32228	32094	214096	17
Shanxi	35063	34876	38075	36768	30575	29780	205137	18
Guangxi	34105	35252	37086	35547	31478	31565	205033	19
Heilongjiang	36540	36234	34924	35779	29583	28504	201564	20
Jiangxi	28492	33747	35540	34926	32603	31731	197039	21
Jilin	35485	35993	33440	32590	27933	29033	194474	22
Guizhou	33143	35926	33789	33970	26544	27875	191247	23
Tianjin	29677	29253	30240	30417	24503	26880	170970	24
Xinjiang	30932	30666	30163	30139	23076	25010	169986	25
Inner Mongolia	26460	29751	31489	31313	25690	25149	169852	26
Gansu	21678	25086	30083	30173	24363	23092	154475	27
Hainan	17782	20975	21115	25411	17221	19293	121797	28
Qinghai	11897	13742	12364	11505	8137	6432	64077	29
Ningxia	5362	7363	9604	11301	8915	8996	51541	30
Tibet	4092	6203	5465	5651	5053	4775	31239	31

The G, H, P, and CV values witnessed from 2016 to 2021 did not change considerably overall (Table 3). G remained between 19.3157 and 19.4636, demonstrating some degree of fluctuation, and there was a yearly decrease between 2016 and 2019, indicating that the spatial distribution index of network attention to hiking gradually increased over this period, but gradually decreased again in 2020 and 2021 under the influence of COVID-19. H remained between 0.0373 and 0.0379 from 2016 to 2021, almost unaffected by COVID-19, indicating a low degree of clustering of network attention, which remained scattered across the 31 provincial-level administrative regions. P slowly increased from 1.4107 in 2016 to 1.5551 in 2019, indicating a gradual concentration of network attention, and then gradually decreased over the following two years to 1.3482 in 2021, indicating that, affected by COVID-19, network attention to hiking remained a dispersed trend. CV gradually decreased from 0.4126 in 2016 to 0.3957 in 2019 and then increased to 0.4176 in 2021, indicating a decrease in spatial variation followed by an increase beyond its original level due to COVID-19. With the introduction in 2020 of travel restrictions, lockdowns, and social distancing norms in response to COVID-19, people’s ability to hike, especially in far-flung locations, will have been greatly affected. This may have resulted in fewer online searches and discussions about hiking destinations that were no longer accessible, affecting network attention.

**Table 3:** Spatial distribution characteristics index of hiking network attention, 2016–2021.

Various indices				
Year	Geographical concentration index G	Herfindahl Index H	Primacy index P	Coefficient of variation CV
2016	19.42907665	0.037748902	1.410747664	0.412572371
2017	19.34626039	0.037427779	1.500235155	0.400326307
2018	19.3529086	0.037453507	1.509048167	0.401321219
2019	19.3156681	0.037309503	1.555102253	0.395720364
2020	19.40400334	0.037651535	1.377432208	0.408897997
2021	19.46363613	0.037883313	1.348183939	0.417591555

#### 3.2.2 Interregional differences

In order to further analyze the statistics on network attention to hiking in different regions of China, we calculated G, H, P, and CV for the various regions (Table 4). From 2016 to 2018, network attention levels gradually increased, with East China’s by far the highest and those of the Northeast and Northwest the lowest. In 2019, the network attention levels of the Central, North, Northeast, Northwest, and Southwest regions all declined, and all regions saw a decline in 2020. By 2021, the East, South, and Central regions recovered from this, but levels in the Central, North, Northeast, Northwest, and Southwest regions continued to decline.

**Table 4:** Spatial distribution characteristic index of hiking network attention.

Year	Index	East	South	Central	North	Northeast	Northwest	Southwest
2016	Attention	319102	130381	125390	190855	112333	104277	157751
	G	38.4814	67.04264	57.78801	46.62564	57.8232	50.57153	49.68793
	H	0.148082	0.449472	0.333945	0.217395	0.334352	0.255748	0.246889
	P	1.030962	2.301539	1.037694	1.408638	1.10312	1.112376	1.448916
	CV	0.1972	0.590267	0.042851	0.294916	0.055288	0.527958	0.484196
2017	Attention	338786	142354	131279	193115	114682	113480	167890
	G	38.36716	67.01365	57.76175	46.32274	57.91148	49.48941	48.84619
	H	0.147204	0.449083	0.333642	0.21458	0.335374	0.24492	0.238595
	P	1.046528	2.443181	1.0028	1.372567	1.17169	1.194254	1.355989
	CV	0.174435	0.589278	0.030428	0.269996	0.078243	0.473921	0.439289
2018	Attention	350444	147428	135041	204299	111772	123496	169318
	G	38.35665	67.08861	57.74614	46.20327	58.13465	49.67957	49.15628
	H	0.147123	0.450088	0.333462	0.213474	0.337964	0.246806	0.241634
	P	1.053597	2.405948	1.038183	1.303326	1.242927	1.36863	1.335688
	CV	0.172808	0.591831	0.01962	0.25956	0.117861	0.483766	0.456256
2019	Attention	350533	150535	134654	201759	109790	122917	168346
	G	38.31752	66.20798	57.74579	46.12475	58.02167	49.2374	49.09268
	H	0.146823	0.43835	0.333458	0.212749	0.336651	0.242432	0.241009
	P	1.03742	2.519959	1.005732	1.261569	1.15769	1.319027	1.341868
	CV	0.166621	0.561292	0.019313	0.25248	0.099772	0.460609	0.45282
2020	Attention	313934	119915	117619	173509	92269	97251	147687
	G	38.21501	66.50065	57.73669	46.65321	57.99203	49.79365	49.55175
	H	0.146039	0.442234	0.333353	0.217652	0.336308	0.247941	0.245538
	P	1.008357	2.262405	1.000736	1.259826	1.174763	1.344662	1.3295
	CV	0.149235	0.571578	0.007587	0.297087	0.09446	0.489595	0.477167
2021	Attention	318345	124241	120104	174019	92655	96878	144940
	G	38.38198	66.14603	57.74318	46.55628	58.00693	50.4961	49.41614
	H	0.147318	0.43753	0.333428	0.216749	0.33648	0.254986	0.244196
	P	1.022947	2.324822	1.022154	1.295666	1.209589	1.333387	1.386715
	CV	0.176702	0.559097	0.016811	0.289385	0.097166	0.524336	0.470082

Three indicators in the East China region declined year by year from 2016 to 2020, namely G, H, and CV, but all increased slowly again in 2021. P, on the other hand, showed volatility, slowly increasing to a peak in 2018, decreasing in 2019 and 2020, and increasing again in 2021. This indicates not only that East China was the region with the highest network attention to hiking, but also that its subregions experienced balanced levels of network attention. South China had the highest values for the four indicators across the seven regions, and all four indicators fluctuated but did not change substantially between 2016 and 2021, indicating the continued unevenness and highly concentrated distribution of network attention to hiking in the five provinces and cities of South China.

All four indicators were also relatively high in Central China and Northeast China, fluctuating slightly between 2016 and 2021 but changing little. These figures show the uneven distribution of network attention to hiking in Central and Northeast China. In North China, moreover, P declined from 1.0376 in 2016 to 1.0007 in 2020 before rebounding in 2021, while CV decreased from 0.2949 in 2016 to 0.2524 in 2019, rose to 0.2971 in 2020, exceeding the 2016 value, and decreased slightly in 2021, indicating the high concentration of network attention to hiking in North China. In the Northwest and Southwest, the four indicators fluctuated during these six years but did not substantially change, and the internal concentration of network attention to hiking across these regions remained high.

#### 3.2.3 Spatial evolution

**Fig 1:**
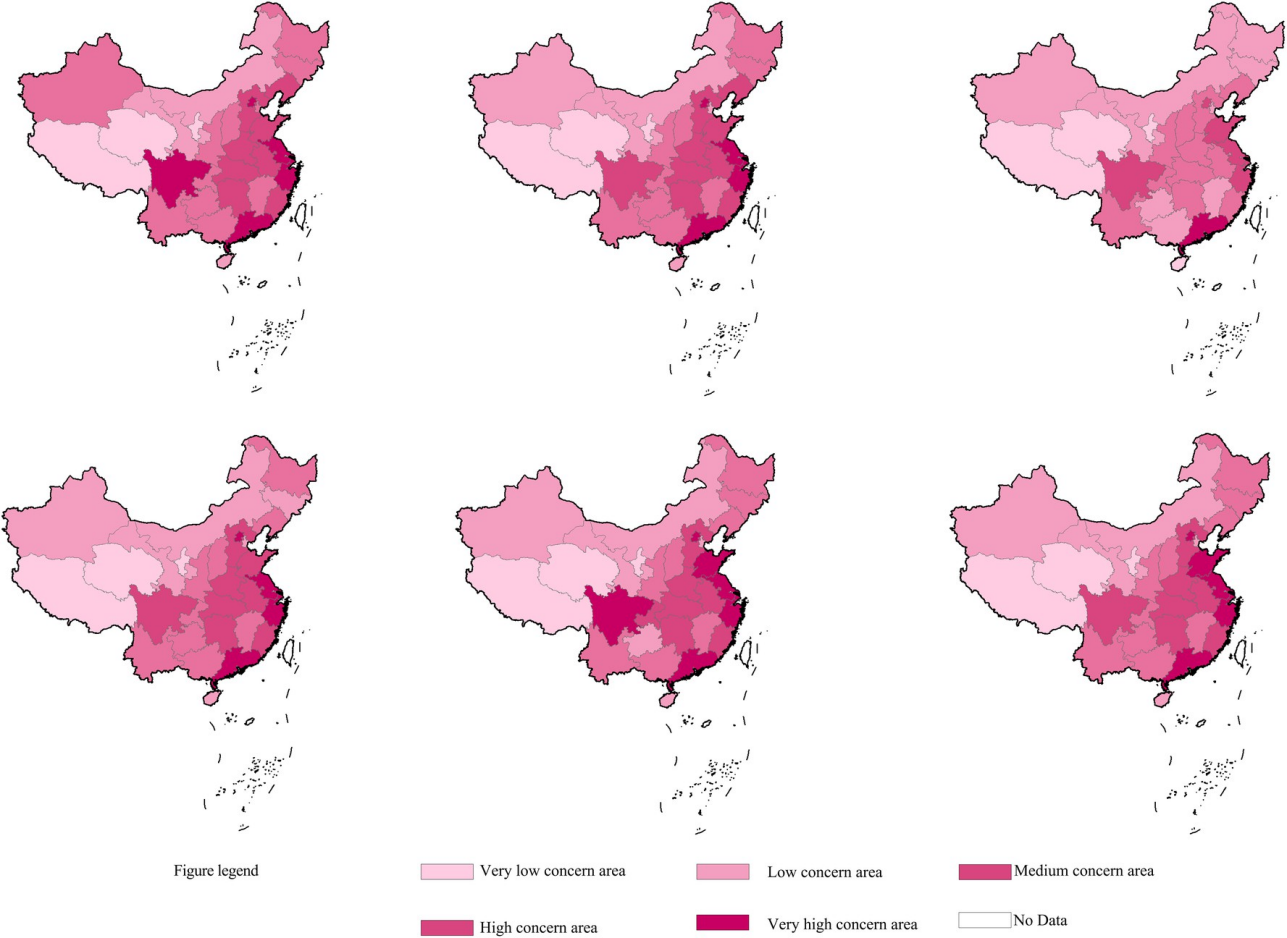
Hierarchical pattern evolution of hiking network attention from 2016 to 2021. Source: The primitive base map from the website of the Ministry of Natural Resources of China (http://bzdt.ch.mnr.gov.cn/) was plotted by ArcGIS 10.2 software (ESRI1), and its Drawing Review Number is GS (2020) 4619.

We used the natural breakpoint grading method in ArcGIS to divide the 31 provincial-level administrative regions directly under the central government into five categories: very low concern area, low concern area, medium concern area, high concern area, and very high concern area. ArcGIS software was then used to spatially visualize network attention to hiking in China from 2016 to 2021, with different color shades indicating the level of network attention in each region(see Fig 1). The results showed that the overall trend of network attention in China’s provinces was relatively stable at a provincial scale, and high concern and very high concern areas were mainly found among the Eastern coastal provinces and Sichuan Province, which was classified as a very high concern area in 2016 and 2020. In 2018, the distribution of network attention across provinces was at its most balanced within the six-year period, with only one very high concern area, while in 2020 and 2021, which were affected by COVID-19, Shandong Province changed from a high concern area to a very high concern area. In terms of interregional differences, while these were present between the Eastern, Central, and Western regions, they remained largely unchanged over the six-year period.

## 4. Analysis of the factors influencing spatial and temporal differences

The objective of this study was to investigate the use of data acquired via search engines, as well as to apply social network analysis to uncover the consumer perspective. It is commonly asserted that conventional models used for forecasting tourism demand, which mostly rely on traditional metrics such as univariate analysis of tourist arrivals, can be enhanced through the use of big data extracted from online sources [4]. This study demonstrates the efficacy of employing social network and semantic analysis techniques on large-scale data obtained from search engines to facilitate the forecasting of visitor arrivals. A significant number of consumers engage in the practice of conducting online searches using search engines before making a final choice to purchase a product or service: The search behavior exhibited by these consumers serves as a manifestation of their genuine interest. Therefore, search engines play a crucial role as a communication medium for businesses to engage with existing and prospective consumers. In order to achieve success in search engine marketing, it is imperative for companies to possess a comprehensive understanding of consumers’ web search activities [27, 65, 66].

Scholars believe that any factor that can influence the needs and access to information of individuals is a factor that affects network attention [8]. Based on existing research [45, 46, 67, 68], combined with the spatial and temporal patterns described above, the factors influencing spatial and temporal differences in network attention to hiking may include level of economic development, degree of network development, population size, and population age structure.

### 4.1 Level of economic development

Higher levels of economic development in a region where Internet users or potential hikers are located lead to higher gross domestic product per capita, fostering better infrastructure and services, greater integration, and greater availability of information. We identified a significant positive correlation between network attention to hiking and the level of economic development in a region (p<0.01; Table 5). One potential reason for this may be that individuals from economically developed areas or high-consumption groups tend to be the main consumers in the tourism market. Consumers’ income, consumption, and purchasing power are determined by their economic level: The more established the economy, the greater the potential desire to pay and the greater the demand for information, which influences network attention levels [19]. Another probable reason is that the higher the disposable income per capita is and the higher the consumption level of the residents of a region, the greater their willingness will be to engage in hiking activities, a pastime particularly influenced by income levels due to its being more expensive than many other tourist activities [23].

**Table 5:** Correlation analysis of factors influencing spatial and temporal.

Variables	Factors	Person Relevance	p-value
Level of economic development	Gross Regional Product	0.866**	0.000
	Gross regional product per capita	0.563**	0.000
Population size	Year-end resident population	0.762**	0.000
Degree of network development	Number of people with Internet access	0.860**	0.000
Population age structure	Population aged 15–64	0.773**	0.000
	Number of people aged 65 and over	0.692**	0.000

Note: ** indicates significant at the 0.05 level.

### 4.2 Degree of network development

The number of Internet users in a region has a direct impact on network attention. The Baidu Index provides a count of Internet searches, which corresponds to the level of network attention, so the more Internet users there are, the more searches there are, and thus the more network attention. We identified a significant positive correlation between Internet penetration rate and network attention to hiking (p<0.01; Table 5). Simultaneously, the higher the degree of Internet informatization, the more information channels potential hikers can access, the more easily information can be accessed, and the higher the network attention.

### 4.3 Population size

Population size was the most fundamental factor in determining the level of network attention. The greater a province’s population, the greater its level of network attention to hiking. The number of Internet users in the information field may better reflect the network behavior of regional Internet users [6]. We identified a significant positive correlation between population size and network attention to hiking (p<0.01; Table 5). The greater the population of a region, the greater the number of network users and potential hikers, and so the higher the level of network attention to hiking in that region.

## 4.4 Population age structure

China’s National Bureau of Statistics and most local statistical offices only count the number of people aged under 15, 15–64, and 65 and over. Based on these data, however, there was a significant correlation between the population of a region aged 15–64, the population aged 65 and over, and the level of network attention to hiking (p<0.01; Table 5). The majority of network users are those between the ages of 15 and 64, as are the majority of hikers. The population will be more dependent on and use the network more frequently the younger it is [6, 69, 70].

## 5. Conclusions and discussion

### 5.1 Conclusions

This study used the Baidu Index to collect the search counts for the keyword “hiking” across a total of 31 provincial-level administrative regions in China from 2016 to 2021 and constructed an index of network attention to hiking, which was analyzed in terms of its spatio-temporal variation characteristics to produce the following conclusions.

First, in terms of temporal distribution, the national network attention index remained fairly stable during 2016–2019, with a somewhat pronounced decline in 2020 due to COVID-19 and a moderate degree of recovery in 2021. Across the six-year period, March, April, and May saw a relatively high degree of network attention to hiking, demonstrating a preference among hikers to engage in hiking activities in the summer.

Second, in terms of spatial distribution, network attention to hiking showed a clear geographical imbalance at the national level, with the index generally decreasing from East to West. Specifically, in the Eastern region, all provinces except for Liaoning Province, Tianjin City, and Hainan Province (which had the lowest index in the region) ranked very highly; in the Central region, Henan, Hebei, Hunan, and Anhui had high levels of network attention; in the Western region, Sichuan had the highest level, ranking sixth in China, far ahead of other Western provinces and cities. From an intraregional perspective, East China had high concentration and a relatively balanced distribution within the region; South China had a highly concentrated and highly unbalanced distribution within the region; Central China, Northeast China, North China, West China and Southwest China all had unbalanced distributions of network attention.

Third, in terms of influencing factors, the level of regional economic development, the degree of network development, and the size of the population directly affected network attention to hiking. This suggests that hikers tend to be distributed in regions with large populations, well-developed economies, high per capita disposable income, and well-developed networks, which makes sense given the higher cost of hiking as a sport.

Fourth, of the four indicators considered, G increased due to COVID-19; H experienced almost no effect; P decreased considerably; and COVID-19 caused an increase in CV.

### 5.2 Discussion

First, in terms of time, network attention to hiking has strong seasonal characteristics. The reason for this is that tourism activities such as hiking are inherently different in off-season and peak season. Network attention to hiking has an obvious “precursor effect” [45]: Hikers need to collect various information in advance before beginning their activity because hiking is potentially dangerous and requires them to consider the weather, transport, supplies, safety, and the difficulty factor of the trail. Information searches are therefore crucial, which leads to a high level of Internet searches and Internet interest in hiking.

Second, in terms of space, network attention to hiking has an obvious geographical imbalance at the national level. In East China, Liaoning Province, Tianjin City, and Hainan Province exhibited low levels of network attention. This may be because Liaoning has an aging population and relatively few young people, while Tianjin City has the smallest population of the four municipalities directly under the central government, and Hainan has the lowest-performing economy in the Eastern region. In the Central region, Henan, Hebei, Hunan, and Anhui exhibited a high level of network attention, possibly because these provinces have relatively large populations. In the Western region, Sichuan had the highest network attention index probably due to its successful economy and large population. Notably, Sichuan’s people are also famous for their enjoyment of leisure activities. East China had a high level of network attention and a relatively balanced distribution within the region, perhaps due to the relatively prosperous economies of its provinces. Network attention to hiking in South China was highly concentrated and strongly unbalanced within the region, probably due to Guangdong Province’s enjoyment of both the most successful economy in China and the largest population. Guangxi and Hainan, conversely, are both economically undeveloped provinces. Central China, Northeast China, North China, West China, and Southwest China all showed unbalanced distributions of network attention probably because they all include provinces with a range of economic development levels. The higher the level of economic development a province has, and the higher its resulting per capita gross domestic product, the more tourism activities are available and the more people engage in activities such as hiking [45, 46].

Third, from the perspective of population characteristics, different regions and different age groups have different levels of interest in hiking. Economically developed areas and high-consumption groups constitute the main consumer groups in the tourism market [45, 46], and hiking is one of the more expensive types of tourism activity, further concentrating its practitioners in economically developed areas and among people with higher incomes.

Fourth, COVID-19 had a noticeable impact on the primacy index, which decreased. The most plausible explanation is that the newly heightened inconvenience of hiking caused the network attention between the first and second province to drop, and the gap between the two thus narrowed. The coefficient of variation, meanwhile, increased, as the negative impact of COVID-19 on the economy made economic differences within provinces and regions more pronounced.

## 6. Implications and limitations

### 6.1 Implications

This research presents one of the first attempts to analyze the network attention to hiking and the factors influencing hiking as a tourism activity in China. Population size and economic development were identified as factors influencing network attention to hiking. The study also provides substantial evidence to support the contention that COVID-19 has hindered the expansion of the Chinese tourism industry. While the effects of the pandemic have continued to evolve, moreover, overall trends in 2021 do not seem to represent decisive signs of recovery.

This study offers destination management organizations several suggestions for effectively interacting with trail hikers. As hiking is a form of sports tourism, hikers not only get in touch with nature but also exercise their bodies while traveling. In China, the USA, and various European countries, increasing numbers of tourists are engaging in hiking activities. With the impact of COVID-19 on hiking having been much lighter than that experienced by other tourism activities, sports tourism stands to become the single most important tourism activity in the post-pandemic era. Moreover, with the generally high income and tourism expenditure of hikers, local governments and companies can use it as a growth point for tourism, and tourist attractions can build more hiking trails and design more hiking activities as a means of attracting more tourists. Finally, network attention can be influenced by Internet celebrities and Internet personalities, so it could be fruitful for organizations to make full use of new media developments in China and actively engage in various marketing efforts to increase online exposure and attention.

### 6.2 Limitations

Although this study provides a new perspective for the study of network attention to hiking in China, it is subject to some limitations that nonetheless suggest avenues for further research. Firstly, the Baidu Index, while it is a reasonable way to analyze levels of network attention, is not fully representative of overall levels of network attention, as ever more consumers are choosing new media such as TikTok to search for information. Secondly, the elements impacting network attention are intricate and multidimensional, and the index system employed in this research may not sufficiently capture these. Thirdly, this study does not integrate online and offline data sources, which could improve the completeness and reliability of the data. In the future, the combination of small-scale, fine-grained analysis and comparative research at different regional levels will offer opportunities to further enrich the depth and breadth of research in this area.
